# A Case of Drug-Resistant Myoclonus Improved by Only Slight Adjustment to the Hemodialysis Setting

**DOI:** 10.7759/cureus.36104

**Published:** 2023-03-13

**Authors:** Ryo Sasaki, Chika Matsuoka, Toru Yamashita, Masaru Kinomura, Koji Abe

**Affiliations:** 1 Department of Neurology, Okayama University, Okayama City, JPN; 2 Department of Neurology, Hiroshima City Hiroshima Citizens Hospital, Hiroshima, JPN; 3 Department of Nephrology, Rheumatology, Endocrinology and Metabolism, Okayama University, Okayama City, JPN; 4 Department of Neurology, National Center of Neurology and Psychiatry, Tokyo, JPN

**Keywords:** dialysis disequilibrium syndrome, involuntary movement, renal failure, myoclonus, hemodialysis

## Abstract

Myoclonus, a rare complication in patients with end-stage renal disease, is typically ameliorated through hemodialysis. The present case concerns an 84-year-old male with chronic renal failure undergoing hemodialysis, presenting involuntary movements in his limbs, which gradually worsened from the initiation of hemodialysis without constant elevation of serum blood urea nitrogen and electrolytes levels. Surface electromyography revealed characteristic findings consistent with myoclonus. He was diagnosed with subcortical-nonsegmental myoclonus related to hemodialysis, and the myoclonus was significantly alleviated after slightly increasing the post-dialysis target weight even though drug treatment was ineffective. This case suggests that drug-resistant myoclonus in patients with renal failure may be improved by adjusting hemodialysis settings, even in cases of atypical dialysis disequilibrium syndrome.

## Introduction

Hemodialysis is one of the treatments for end-stage renal disease, and over 330,000 patients in Japan receive hemodialysis in one year [1]. Patients with renal disease sometimes show neurological symptoms such as stroke, headache, cognitive impairment, and involuntary movements, including myoclonus [2]. In general, the cause of myoclonus is variable such as myoclonic epilepsy, encephalitis, neurodegenerative diseases like Creutzfeldt-Jakob disease, drugs, and uremia or other metabolic disorders [3]. Myoclonus caused by uremia is usually improved by hemodialysis paralleled with the decrease of serum blood urea nitrogen level. However, there were no previous reports of myoclonus worsened by hemodialysis. Here, we report a unique case of end-stage renal disease patient showing drug-resistant myoclonus two years after induction of dialysis, which was paradoxically worsened by hemodialysis and improved by adjusting the hemodialysis settings.

## Case presentation

**Figure 1:**
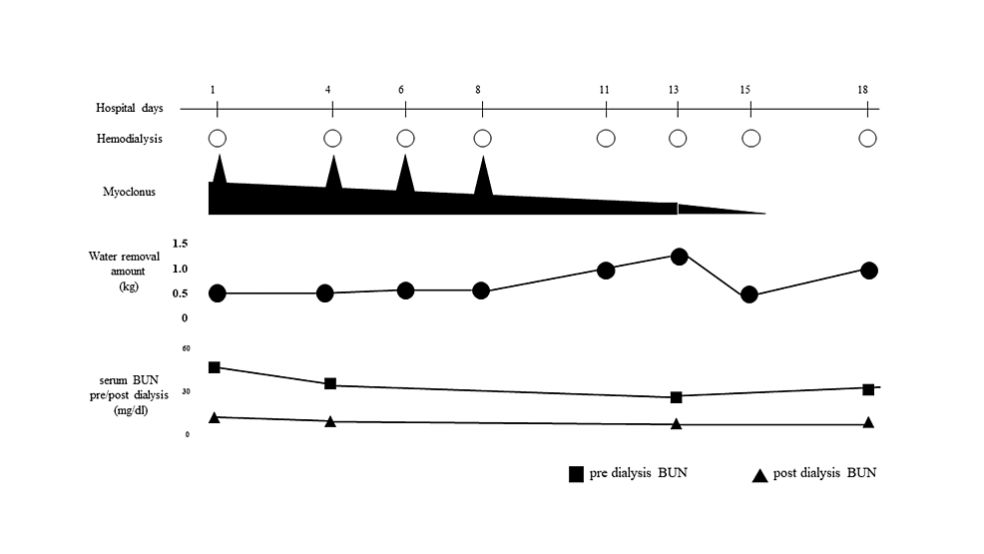
Clinical course of the present case. Myoclonus worsened during hemodialysis on 1, 4, 6, and 8 hospital days (upper panels). With decreasing the total water removal amount on day 11, the myoclonus disappeared on day 13. Serum BUN at pre/post dialysis was slightly decreased (lower panels). BUN: Blood urea nitrogen.

An 84-year-old man had been treated for type 2 diabetes mellitus and hyperuricemia from the age of 45. He showed chronic renal failure from age 64, and hemodialysis was induced at age 82. An involuntary movement of the limbs and trunk, evident during hemodialysis, was found at age 84. This involuntary movement was gradually exacerbated from the start of hemodialysis and sustained for 3-4 hours afterward. On non-dialysis days, the frequency of involuntary movement was relatively rare. The involuntary movement remained even after taking 0.5 mg of clonazepam; thus, he was admitted to our hospital (Figure 1). On admission, he took 20 mg of febuxostat, 100 mg of aspirin, 5 mg of linagliptin, 40 mg of furosemide, and 750 mg of bixalomer capsules. Furthermore, the hemodialysis regimen remained unchanged. Our institution's protocol for hemodialysis involves three sessions per week, with each session lasting four hours. Dalteparin was used as an anticoagulant medication, administered at an initial dose of 750 units, followed by a continuous dose of 500 units per hour.

**Figure 2:**
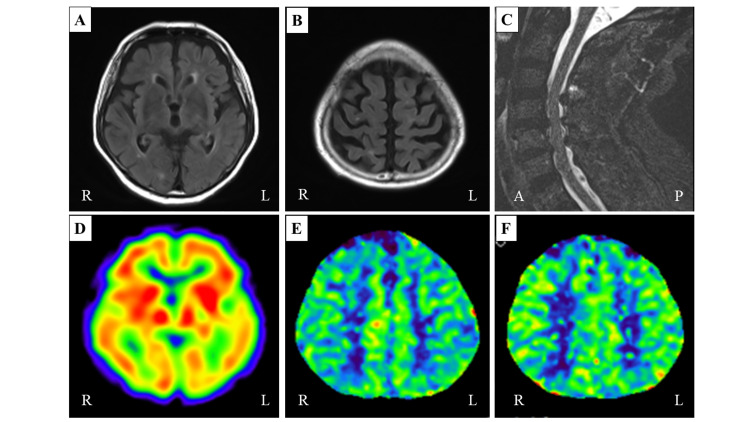
Clinical examinations of the patient. (A, B) Brain MRI showing no abnormality. (C) Spinal MRI showing mild cervical spondylosis. (D) IMP-SPECT showing no blood flow decrease in the brain cortex. (E-F) There is no change in cerebral blood flow on the perfusion CT between pre-dialysis (E) and post-dialysis (F). IMP: N-isopropyl[123I]-p-iodoamphetamine; SPECT: Single photon emission computed tomography.

He was 158.6 cm tall and weighed 62.6 kg. His blood pressure was 100/64 mmHg with a body temperature of 36.5 °C. Neurological examination revealed mild cognitive dysfunction with 26/30 in mini-mental state examination (MMSE), 24/30 in Hasegawa dementia scale-revised (HDS-R), and hyporeflexia in all extremities (A/E). Muscle strength, sensory, cerebellar, and autonomic systems were all normal. Quick and non-rhythmical involuntary movements were shown in A/E and trunk, especially in the right upper extremities (U/E), which decreased when he talked. Serum analyses showed an elevation of blood sugar (251 mg/dl, normal 73-109 mg/dl), hemoglobin A1c (HbA1c) (7.1%, normal 4.9-6.0 %), blood urea nitrogen (BUN) (48.8 mg/dl, normal 8-20 mg/dl), and creatinine (8.86 mg/dl, normal 0.65-1.09 mg/dl). A cerebral spinal fluid study showed normal cell counts (0/µl), moderately elevated protein (102 mg/dl), and a normal IgG index (0.49, normal ≤0.60). Whole-body CT showed no evident feature of malignancy. A brain MRI showed no lesions or signal change in the bilateral basal ganglia and cortex on fluid-attenuated inversion recovery (FLAIR) images (Figures 2A-2B) and diffusion-weighted images (DWI, data not shown). MRI of the cervical spine showed mild spinal canal stenosis without signal change of the cervical medulla (Figure 2C). Brain single photon emission computed tomography (SPECT) showed no increase or decrease of blood flow in the basal ganglia or cortex (Figure 2D), and there was no cerebral blood flow change compared to before and after dialysis on perfusion CT (Figures 2E-2F).

**Figure 3:**
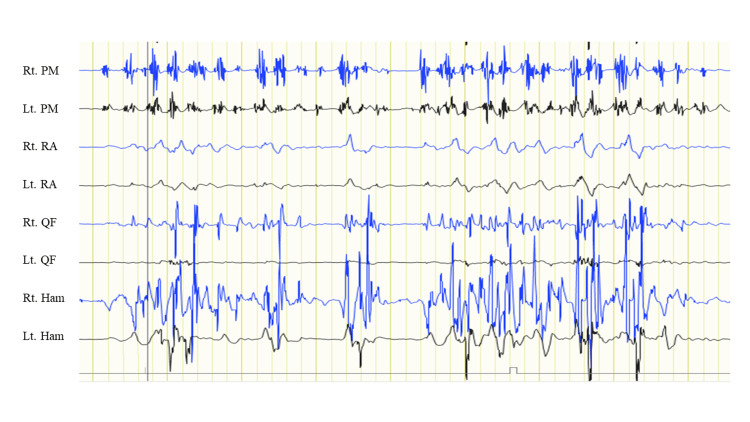
Surface electromyography. Surface electromyography in bilateral pectoralis major (PM), rectus abdominus (RA), quadriceps femoris (QF), and hamstrings (Ham) showing muscles co-contraction around 100 ms of duration spreading from unilateral U/E to A/E.

Electroencephalogram (EEG) during the symptomatic state showed normal findings, and somatosensory evoked potentials (SEP) to the right median nerve stimulation at the wrist showed no giant SEP. Surface electromyography in bilateral pectoralis major (PM), rectus abdominus (RA), quadriceps femoris (QF), and hamstrings (Ham) showed partially rhythmic groups of discharges with 80-100 ms duration started from Rt U/E and spread to Lt. U/E, trunk, and bilateral lower extremities (L/E) (Figure 3).

He was diagnosed with myoclonus related to hemodialysis. Since myoclonus was observed during hemodialysis on days 1, 4, 6, and 8 after admission, we increased the post-dialysis target weight from 61.5 kg to 62.0 kg from day 11 to avoid radical changes in circulating plasma volume. After the target weight change, the myoclonus significantly decreased and finally disappeared on day 13 (Figure 1).

## Discussion

This is the case report of a transient subacute myoclonus patient during hemodialysis improved by increasing the post-dialysis target weight. After two years of hemodialysis history without any neurological symptoms, the patient showed myoclonus during hemodialysis (Figure 1). No pathological findings were shown in the brain and cervical spinal MRI (Figures 2A-2C). No blood flow changes were even compared before and after the hemodialysis (Figures 2E-2F), and surface electromyography showed the characteristic of myoclonus (Figure 3). Myoclonus is a prominent form of spasmodic movement characterized by brief, sudden contractions or relaxations of muscle groups [3]. It is often classified according to its clinical presentation and examination results, such as cortical, cortical-subcortical, subcortical-nonsegmental, segmental, and peripheral myoclonus [4]. In the present case, we suspected that the involuntary movement could be classified as subcortical-nonsegmental myoclonus because it was characterized by muscle co-contraction around 100 ms of duration spreading from one U/E to the other lower extremity, with normal results on somatosensory evoked potentials and electroencephalography.

Several previous reports of patients with subacute myoclonus coincidence of renal failure revealed that the cause of myoclonus were drugs such as gabapentin [5], pregabalin [6], and corticosteroid [7], and cervical lesions, including severe cervical spondylosis [8], and uremia [9]. In the present case, the patient did not take the causative agent causing myoclonus nor had severe cervical lesions. Moreover, drug-induced myoclonus can generally occur on non-dialysis days due to drug administration, unlike dialysis-related myoclonus. If the myoclonus was caused by uremia, the myoclonus should have improved after the start of dialysis. However, it worsened after the start of dialysis and improved by reducing the amount of water removed by hemodialysis. Thus, in the present case, uremia was not just a cause of myoclonus by itself. After excluding other potential causes, we determined that the dialysis disequilibrium syndrome was the likely etiology of the patient's myoclonus. This syndrome, which is characterized by central nervous system edema, is commonly observed in individuals undergoing initial dialysis or those with a prior history of neurological disorders [10]. However, it can also occur in patients who have been on hemodialysis for an extended period of time. The fact that the patient's symptoms were exacerbated by hemodialysis and improved with only minimal adjustments to the dialysis setting suggests that the myoclonus may be related to the concentration of substances in the blood or total plasma volume. The patient may have underlying factors that contribute to the hypersensitivity of their central nervous system to the blood-brain concentration gradient. Further studies, including detailed analysis of blood and spinal fluid during dialysis, may be necessary to understand the pathophysiology of this case. The present case indicated that myoclonus in the patient with renal failure could be improved by only slightly adjusting the post-dialysis target weight even if the patient's background was atypical of the dialysis disequilibrium syndrome. This may provide the treatment option for unexplained intradialytic myoclonus.

## Conclusions

We report a unique case of end-stage renal disease patient showing drug-resistant myoclonus, which was worsened by hemodialysis and improved by adjusting the hemodialysis settings very slightly. The present case provides new insights into how the hemodialysis-related myoclonus could be treated without additional drugs by only adjusting the post-dialysis target weight, even if the patient with a long history of stable hemodialysis settings.
